# Punicalagin, an active component in pomegranate, ameliorates cardiac mitochondrial impairment in obese rats via AMPK activation

**DOI:** 10.1038/srep14014

**Published:** 2015-09-15

**Authors:** Ke Cao, Jie Xu, Wenjun Pu, Zhizhong Dong, Lei Sun, Weijin Zang, Feng Gao, Yong Zhang, Zhihui Feng, Jiankang Liu

**Affiliations:** 1 Center for Mitochondrial Biology and Medicine, Frontier Institute of Science and Technology, Xi’an Jiaotong University, Xi’an, 710049, China; 2 The Key Laboratory of Biomedical Information Engineering of Ministry of Education, School of Life Science and Technology, Xi’an Jiaotong University, Xi’an, 710049, China; 3 Nestlé Research Center Beijing, Beijing, 100095, China; 4 Department of Pharmacology, School of Medicine, Xi’an Jiaotong University, Xi’an, 710049, China; 5 Department of Aerospace Medicine, Fourth Military Medical University, Xi’an, 710032, China; 6 Tianjin Key Laboratory of Exercise Physiology and Sports Medicine, Tianjin University of Sport, Tianjin, 300381, China

## Abstract

Obesity is associated with an increasing prevalence of cardiovascular diseases and metabolic syndrome. It is of paramount importance to reduce obesity-associated cardiac dysfunction and impaired energy metabolism. In this study, the activation of the AMP-activated protein kinase (AMPK) pathway by punicalagin (PU), a major ellagitannin in pomegranate was investigated in the heart of a rat obesity model. In male SD rats, eight-week administration of 150 mg/kg pomegranate extract (PE) containing 40% punicalagin sufficiently prevented high-fat diet (HFD)-induced obesity associated accumulation of cardiac triglyceride and cholesterol as well as myocardial damage. Concomitantly, the AMPK pathway was activated, which may account for prevention of mitochondrial loss via upregulating mitochondrial biogenesis and amelioration of oxidative stress via enhancing phase II enzymes in the hearts of HFD rats. Together with the normalized expression of uncoupling proteins and mitochondrial dynamic regulators, PE significantly prevented HFD-induced cardiac ATP loss. Through *in vitro* cultures, we showed that punicalagin was the predominant component that activated AMPK by quickly decreasing the cellular ATP/ADP ratio specifically in cardiomyocytes. Our findings demonstrated that punicalagin, the major active component in PE, could modulate mitochondria and phase II enzymes through AMPK pathway to prevent HFD-induced cardiac metabolic disorders.

As a leading cause of preventable death worldwide, obesity has increased in prevalence in both adults and children and has become one of the most serious public health problems^1^. Previous studies suggest that obesity is closely associated with cardiovascular disorders, including cardiac dysfunction, which is assumed to be the consequence of adaption to the oversupply of substrates such as long-term exposure to a high-fat diet (HFD)^2^,^3^. Increased body weight gain causes the heart to switch from fatty acid burning to sugar burning with the consequence of fat accumulation around the heart, which eventually induces cardiac impairment and increases the risk of myocardial infarction^4^,^5^.

In addition to fat accumulation, several other risk factors such as oxidative stress^6^,^7^ and mitochondrial dysfunction^8^ have also been reported to be closely associated with obesity-induced cardiac metabolic disorders and impairment. Previous study indicated that excessive fat accumulation could amplify ROS generation and establish oxidative stress and morphological changes, which eventually results in heart injury^9^. HFD could result in both a decrease in the mitochondrial quinine pool and a profound modification in the composition of mitochondrial lipids, leading to the inhibition of fatty acid oxidation and increased mitochondrial ROS production^10^. AMP-activated protein kinase (AMPK), the major cellular energy sensor, was recently identified as master regulator of mitochondrial biogenesis^11^,^12^, and its activity was suppressed by HFD in multiple tissues including white adipose tissue, heart, and liver^13^. HFD induced AMPK activity loss was reported to enhance cardiomyocyte death during myocardial ischemia^14^. The deficiency of AMPK could exaggerate HFD induced cardiac hypertrophy and contractile dysfunction^15^. While, the knowledge on AMPK regulated mitochondrial biogenesis in HFD heart is limited and requires further investigation. In addition to mitochondrial biogenesis, recent study suggested that AMPK could modulate oxidative stress through NF-E2 related factor (Nrf2) mediated phase II antioxidant enzymes, and the potential crosstalk has been reported in *Caenorhabditis elegans*^16^, human endothelial cells^17^ and mammalian inflammatory systems^18^. In our previous study, we observed that AMPK could coordinate both peroxisome proliferator-activated receptor gamma coactivator-1 alpha (PGC-1α) mediated mitochondrial biogenesis and Nrf2 mediated phase II enzymes to reduce oxidative stress for neuroprotective effect^19^. Here we proposed that disruption of AMPK-PGC-1α/Nrf2 cascades might be the major contribution to HFD-induced oxidative stress and cardiac impairment.

*Punica granatum*, commonly known as pomegranate, have become increasing popular because of their important biological actions^20^, including cardiovascular protection^21^. Pomegranate juice has been demonstrated to improve the lipid profiles in diabetic patients with hyperlipidemia^22^. Pomegranate flower extract was reported to improve cardiac lipid metabolism and diminish cardiac fibrosis in diabetic rats^23^,^24^. Of the polyphenols found in pomegranate, punicalagin (PU), the most abundant ellagitannin with the highest molecular weight, has been shown to have antioxidant and anti-inflammatory bioactivities^25^,^26^. However, the contributions of PU in pomegranate associated cardiac benefits remain largely unknown. In current study, we investigate the potential effects and underlying mechanism of PU on HFD-induced cardiac impairment, with a specific focus on the AMPK-PGC-1α/Nrf2 cascade.

## Results

### Obesity associated heart metabolic changes

**Figure 1:**
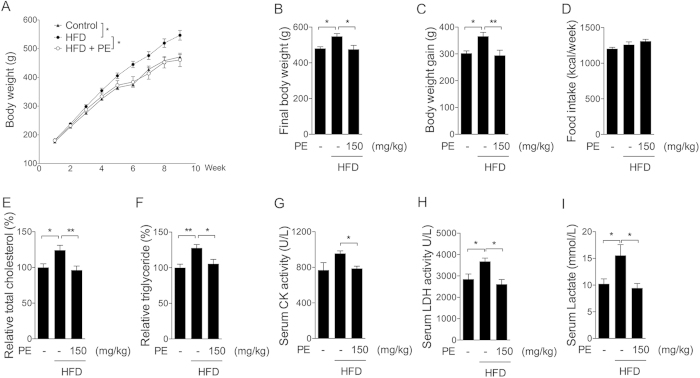
Effect of PE and HFD on obesity-associated metabolic changes in the heart. SD rats in the HFD groups were administered either saline or PE (150 mg/kg/day) for 8 weeks. (**A**) Weekly body weight. (**B**) Final body weight. (**C**) Body weight gain. (**D**) Food intake. (**E**) Triglyceride and (**F**) cholesterol in heart tissues. (**G**) CK activity, (**H**) LDH activity and (**I**) lactate content in rat serum. The values are presented as the means ± S.E.M. (n = 10); *p < 0.05, **p < 0.01.

An HFD rat model was used to investigate how obesity affects cardiac metabolism and potential benefits of PU. Obesity was induced by a HFD over an 8-week period. Since large amount of 100% PU for animal supplement was not available, natural PE containing 40% PU was administrated through oral gavage at a dosage of 150 mg/kg/day during the HFD period. As shown in Fig. 1A–C, the HFD significantly increased body weight and body weight gain, which was efficiently reduced by PE. No significant changes were observed among the three groups regarding food intake (Fig. 1D). It is interesting that PE showed no effect on HFD induced perirenal fat and epididymal fat increase (Fig. S1). Heart tissue analysis showed higher triglyceride and cholesterol levels in the HFD group, which were normalized by PE supplement (Fig. 1E,F). Serum enzyme activities of creatine kinase (CK) and lactate dehydrogenase (LDH) are usually considered markers of cardiac function, and their activities would be more than 10 times higher in cardiac dysfunction rats^27^,^28^. In current HFD feeding, only slight increases were observed on serum CK and LDH activities as well as LDH product lactate, which all were sufficiently decreased by PE supplement (Fig. 1G–I).

### AMPK/ACC pathway in heart

**Figure 2:**
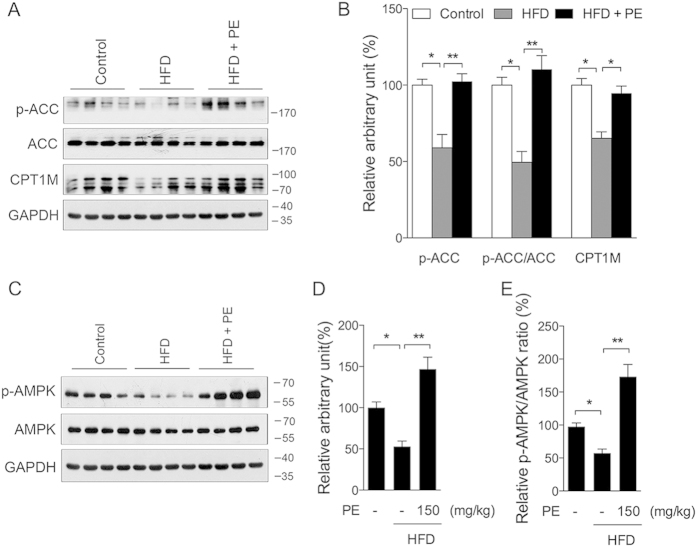
Effect of PE and HFD on the AMPK/ACC pathway. Protein samples were prepared from heart tissue. (**A**) Western blot of p-ACC, ACC, and CPT1M; (**B**) arbitrary unit analysis. (**C**) Western blot of p-AMPK, and AMPK; (**D**) arbitrary unit analysis of p-AMPK; (**E**) p-AMPK/AMPK ratio. The values are presented as the means ± S.E.M. (n = 10); *p < 0.05, **p < 0.01.

Following increased lipid accumulation in the heart tissue, we further investigated the changes of the AMPK pathway, which regulates beta-oxidation and plays a major role in energy homeostasis. As shown in Fig. 2A,B, phosphorylation of ACC, a downstream target of AMPK, was significantly lower in the HFD group and was sufficiently increased by PE. CPT1M, the fatty acid transporter, showed similar changes as p-ACC (Fig. 2A,B). Consistent with these data, the HFD sharply decreased the level of p-AMPK (Fig. 2C,D), and the PE supplement sufficiently promoted the level of p-AMPK as well as the p-AMPK/AMPK ratio (Fig. 2C–E).

### AMPK/ACC activation in cardiomyocytes

**Figure 3:**
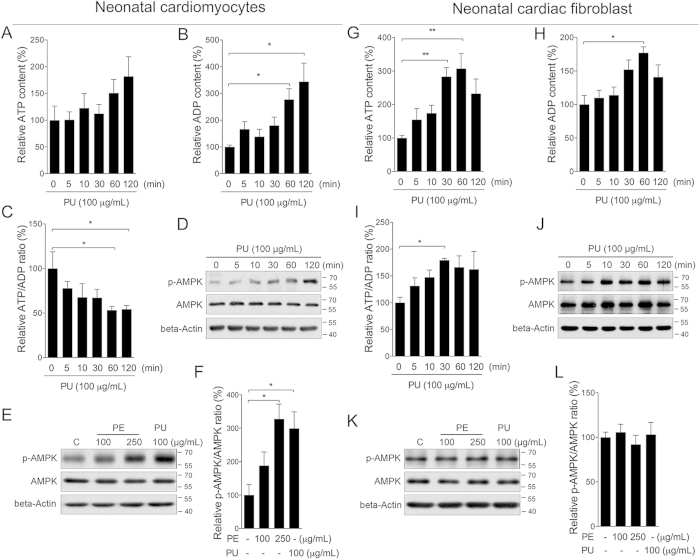
PU activates the AMPK pathway specifically in cardiomyocytes. Neonatal cardiomyocytes and cardiac fibroblasts were isolated from newborn SD rats. Cells were treated with PU at 100 μg/mL for indicate time, cellular ATP, ADP, ATP/ADP ratio, and AMPK phosphorylation was analyzed in neonatal caridomyocytes (**A–D**) and fibroblasts (**G–J**) Then cells were treated with PE at 100, 250 μg/mL and PU at 100 μg/mL for 120 min, AMPK phosphorylation and ratio of p-AMPK/AMPK was analyzed in both caridomyocytes (**E,F**) and fibroblasts (**K,L**). The values are presented as the means ± S.E.M. (n = 4); *p < 0.05, **p < 0.01.

**Figure 4:**
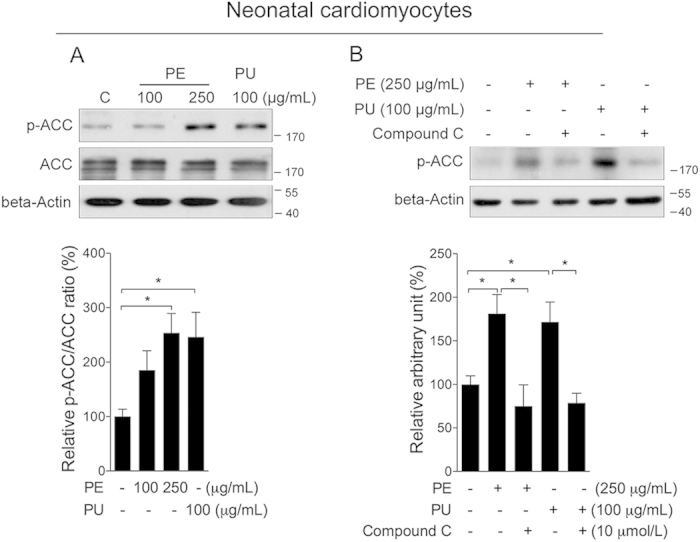
PU induces ACC phosphorylation through AMPK activation in cardiomyocytes. (**A**) Neonatal cardiomyocytes were treated with either PE at 100 and 250 μg/mL or PU at 100 μg/mL for 2 h for analysis of p-ACC and ACC. (**B**) Cells were treated with PE or PU either in the presence or absence of compound C for analysis of p-ACC. The values are presented as the means ± S.E.M. (n = 4); *p < 0.05, **p < 0.01.

To explore whether PU is the major active compound activating AMPK, we developed cellular tests with neonatal cardiomyocytes and fibroblast cells. A time dependent treatment of PU at 100 μg/mL did not show significant effect on ATP level in cardiomyocytes (Fig. 3A), while sharply increased cellular ADP content (Fig. 3B), creating a decreased ATP/ADP ratio (Fig. 3C). Time dependent increase of p-AMPK was observed following PU treatment (Fig. 3D). Further treatment with PE also induced significant phosphorylation of AMPK in neonatal cardiomyocytes. Interestingly, PE at 250 μg/mL showed similar induction as PU at 100 μg/mL (Fig. 3E,F). In neonatal cardiac fibroblast, a time dependent treatment of PU at 100 μg/mL sharply increased ATP level (Fig. 3G), as well as ADP level (Fig. 3H). Since the increase in ATP level was much higher than ADP, PE treatment created an increase ATP/ADP ratio (Fig. 3I). And no significant changes of p-AMPK were observed following PU or PE treatment (Fig. 3J–L). Since ellagic acid (EA) is known as another major compound in nature pomegranate, and PU could be converted to EA through hydrolysis, we also analyzed EA on neonatal cardiomyocytes and found no significant AMPK activation (Fig. S2). These results suggested that PU could be a major active compound of PE, activating the AMPK pathway by decreasing the ATP/ADP ratio. As a major downstream target of AMPK, we found that phosphorylation of ACC was sharply increased after either PE or PU treatment in cardiomyocytes (Fig. 4A). Inhibition of AMPK activity by the specific inhibitor compound C efficiently abolished the effect of PE and PU on ACC phosphorylation (Fig. 4B). Similar with AMPK phosphorylation, PE and PU showed no significant effect on ACC phosphorylation in neonatal cardiac fibroblasts (Fig. S3).

### Mitochondrial biogenesis in cardiomyocyte

**Figure 5:**
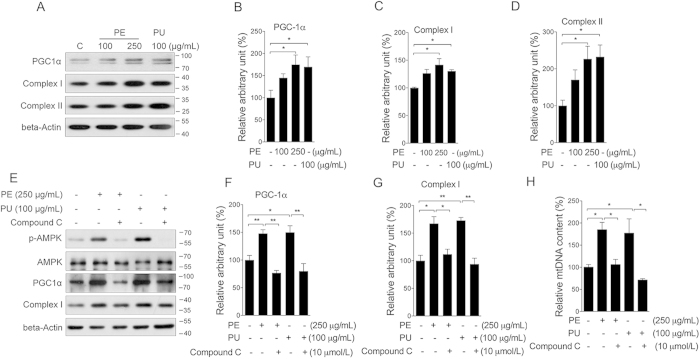
PU induces mitochondrial biogenesis via AMPK pathway activation. Neonatal cardiomyocytes were treated with either PE at 100 and 250 μg/mL or PU at 100 μg/mL for 24 h, protein levels of PGC-1α, complex I, and II were analyzed by western blot (**A**) arbitrary unit was analyzed for PGC-1α (**B**) complex I (**C**) and complex II (**D**) Cells were then treated with PE or PU for 24 h either in the presence or absence of compound C, protein levels of p-AMPK, AMPK, PGC-1α and complex I were analyzed by western blot (**E**) arbitrary unit was analyzed for PGC-1α (**F**) and complex I (**G**) mitochondrial DNA copy number was analyzed by real-time PCR (**H**). The values are presented as the means ± S.E.M. (n = 4); *p < 0.05, **p < 0.01.

**Figure 6:**
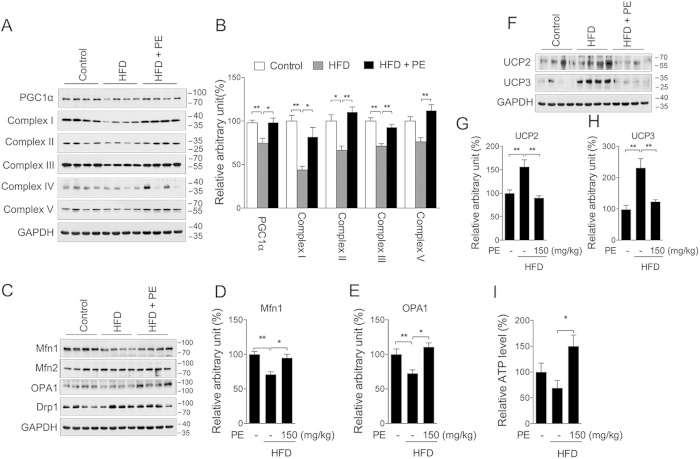
PE promotes mitochondrial remodeling in hearts of rats fed a HFD. Extracts were prepared from the rat heart to assess total protein content. The mitochondrial content was assessed by measuring PGC-1α and the mitochondrial complexes (**A**) western blot image; (**B**) arbitrary units analysis). The mitochondrial dynamic status was assessed by measuring the Mfn1, Mfn2, OPA1 and Drp1 protein expression levels (**C**) western blot image; arbitrary units analysis of (**D**) Mfn1 and (**E**) OPA1). The UCP2 and UCP3 protein levels (**F**) western blot image; arbitrary units analysis of (**G**) UCP2 and (**H**) UCP3) as well as the tissue ATP content (**I**) were also analyzed. The values are presented as the means ± S.E.M. (n = 10); *p < 0.05, **p < 0.01.

Mitochondria are critical organelles for cellular metabolism, including lipid beta-oxidation and ATP production. Previous studies have indicated that AMPK could activate PGC-1α to induce mitochondrial biogenesis^29^. Following phosphorylation of AMPK, we then investigated PU’s effect on mitochondria. As shown in Fig. 5, both PE and PU significantly increased the protein level of PGC-1α (Fig. 5A,B), mitochondrial complex I (Fig. 5A,C), and complex II (Fig. 5A,D) in neonatal cardiomyocytes. No significant changes were observed in cardiac fibroblasts (Fig. S3). The addition of compound C showed sufficient inhibition on the induction of PGC-1α (Fig. 5E,F) and mitochondrial complex I expression (Fig. 5E,G), as well as mitochondrial DNA copy number (Fig. 5H), suggesting that PU could induce mitochondrial biogenesis by activating AMPK in cardiomyocytes. In the heart tissue of HFD rats, we observed decreased protein levels of PGC-1α, mitochondrial complex I, II, III, and V, all of which were sufficiently improved by PE supplement (Fig. 6A,B). As key regulators of mitochondrial fusion, Mfn1 and OPA1 were decreased in the HFD group and increased in the PE supplement group, whereas Mfn2 and the mitochondrial fission related protein Drp1 were unchanged among the three groups (Fig. 6C–E). UCP2 and UCP3, which are known to decouple mitochondrial oxidative phosphorylation from ATP synthesis, were increased in the HFD group and restored to normal levels in the PE supplement group (Fig. F–H). ATP content in heart tissue was significantly increased in the PE group compared to the HFD group (Fig. 6I). These results indicated that PE could normalize cardiac mitochondria metabolism that was disrupted by HFD, and promoting AMPK activity potentially played major contribution.

### Phase II enzymes activation and oxidative damage

**Figure 7:**
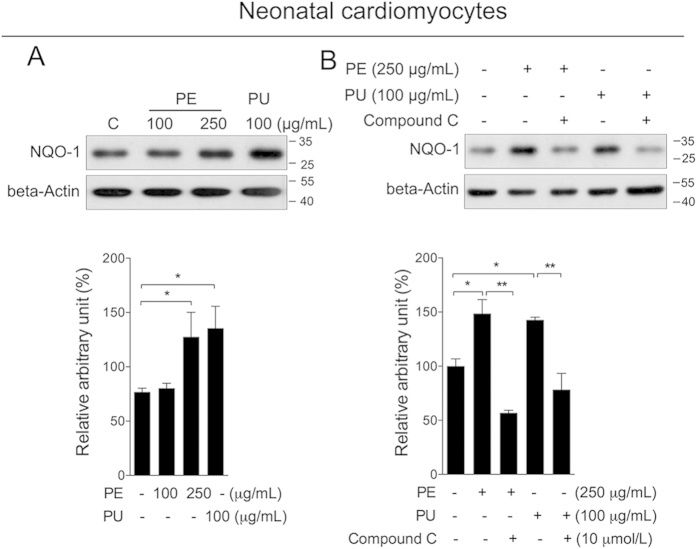
PU activates phase II enzymes through the AMPK pathway. (**A**) Neonatal cardiomyocytes were treated with either PE at 100 and 250 μg/mL or PU at 100 μg/mL for 24 h, protein level of NQO-1 was analyzed by western blot. (**B**) Cells were then treated with PE or PU for 24 h either in the presence or absence of compound (**C**) protein levels of NQO-1 was analyzed by western blot. Values are means ± S.E.M. (n = 4); *p < 0.05, **p < 0.01.

**Figure 8:**
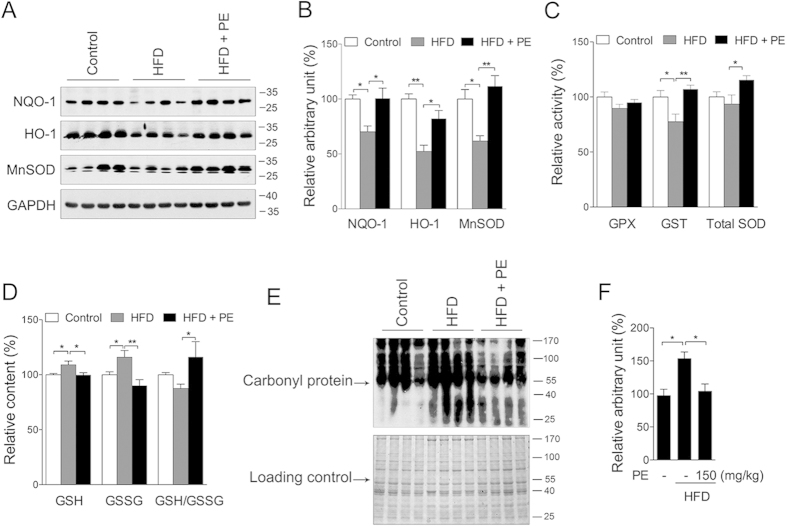
PE eliminates HFD-induced protein oxidation. Total protein extracts were prepared from the rat heart. Protein levels of phase II enzymes NQO-1, HO-1, and MnSOD were analyzed by Western blot (**A**) western blot image; (**B**) arbitrary unit analysis). (**C**) Activity of GPX, GST, and total SOD. (**D**) GSH, GSSG, and GSH/GSSG level. The carbonyl protein levels (an indicator of protein oxidation) were analyzed by western blot (**E**) western blot image; (**F**) arbitrary unit analysis). The values are presented as the means ± S.E.M. (n = 10); *p < 0.05, **p < 0.01.

Previous studies have indicated that fat accumulation and cardiac injury are closely associated with oxidative stress^9^,^30^, which may also result from either mitochondrial dysfunction or suppression of the antioxidant system. To explore the effect of PU on phase II enzymes and oxidative stress, we first measured NQO-1, which was used as marker of all phase II enzymes. Similar to mitochondrial biogenesis, NQO1 was increased by PE and PU in neonatal cardiomyocytes (Fig. 7A), and this effect was abolished by AMPK inhibition (Fig. 7B). Consistently, the induction of NQO-1 by PU or PE was not observed in fibroblasts (Fig. S3). Further analysis of the heart tissue showed that the phase II enzymes NQO-1, HO-1 and MnSOD were significantly decreased in the HFD group and increased in the PE group (Fig. 8A,B). The HFD also reduced GST activity, whereas the GPx and total SOD activities were unaffected; and treatment with PE sufficiently improved the GST and total SOD activities compared to the HFD group (Fig. 8C). Interestingly, GSH, the most abundant antioxidant in living cells, was increased in the HFD group and decreased in PE group (Fig. 8D). Similar changes were also observed on the GSSG level, and the ratio of GSH/GSSG was unchanged in the HFD group compared to the control group, whereas PE treatment significantly increased the ratio of GSH/GSSG (Fig. 8D). The presence of carbonyl protein, an indicator of protein oxidation, was analyzed and observed to be increased in the HFD group and efficiently decreased by PE treatment (Fig. 8E,F). Taken together, these data suggest that PU could activate phase II enzymes through AMPK and ameliorate oxidative damage in the hearts of rats fed a HFD.

## Discussion

Obesity is reported to be closely associated with the onset and development of cardiac hypertrophy and compromised myocardial contractile function, of which the precise mechanisms remain poorly understood^31^. To facilitate studies, long-term HFD feeding or combined challenges have been developed to induce significant cardiac dysfunction in animal models. In mice, HFD was reported to induce cardiac hypertrophy and fibrosis after 11 months feeding^32^, and cardiac contractile dysfunction was reported after 20 weeks HFD feeding^33^ or 16 weeks HFD feeding with three days ethanol challenge^34^. While in rats, 4 weeks HFD feeding followed by one week streptozontocin (STZ) injection would induce cardiac dysfunction after another 7 weeks^35^. HFD feeding for 20 weeks was reported to increase myocardial infarct sizes in Wistar rats^36^. While in current study, we used only 8 week HFD feeding to study the early changes of cardiac tissue with a focus on mitochondria, thereby providing potential targets and pathway that accounts for the benefits of pomegranate.

PU, a major ellagitannin found in pomegranate extract, has been shown to have antioxidant and anti-inflammatory bioactivities^25^,^26^. A toxicity evaluation indicated that 6% PU content in the rat diet (4.8 g of PU/kg/day) over 37 days did not induce any tissue alterations^37^. We have previously shown that following administration of 150 mg/kg PE by oral gavage, the serum PU concentration increased in a time-dependent manner and reached 17.5 μg/mL at 2 h, which suggested that PU could circulate throughout the body and exert its potential effects^38^. It is interesting that PE showed no significant effect on adipose tissue increase while reducing the whole body weight (Fig. S1). As reported previously^38^, PE supplement had no effect on total energy intake comparing to the HFD control, indicating that the decrease on body weight by PE was not due to the reduction of energy intake. Moreover, PU could significantly reduce the lipid deposit in liver through increasing mitochondria function^38^, which was consistent with current observation in heart tissue, we thereby speculate that PU could efficiently promote energy expenditure in mitochondria enriched tissue, contributing to the whole body weight loss under HFD condition.

AMPK is a well-known metabolic master regulator whose activation is mediated by rising AMP and falling ATP. Once activated, AMPK will promote ATP-producing catabolic pathways like fatty acid oxidation and glycolysis, and inhibit ATP-consuming anabolic pathways including lipogenesis. Acetyl-CoA carboxylase (ACC) is a rate-determining enzyme for the synthesis of malonyl-CoA, a critical substrate for both fatty acid biosynthesis and a potent inhibitor of fatty acid oxidation. Through phosphorylation of ACC, AMPK can inhibit ACC activity and thereby decrease fatty acid synthesis^39^,^40^. A deficiency in AMPK was reported to exacerbate HFD-induced cardiac hypertrophy and contractile dysfunction as well as aging-induced myocardial contractile dysfunction^15^,^41^. In our study, the HFD significantly increased body weight gain accompanied by increased cardiac triglyceride and cholesterol levels. Further analysis revealed decreased phosphorylation of AMPK and ACC, suggesting the potential activation of fatty acid biosynthesis. CPT1, which is responsible for transferring long-chain fatty acids into mitochondria for beta-oxidation, was also decreased in HFD rats, suggesting decreased fatty acid oxidation. We assumed that activated fatty acid biosynthesis and decreased oxidation might contribute to cardiac lipid accumulation. PE supplementation during the HFD feeding could sufficiently ameliorate those metabolic changes. The robust increase of p-AMPK in the PE feeding group indicated that PE might directly act on the AMPK pathway. Therefore, we used neonatal cardiomyocytes, and fibroblasts to perform *in vitro* experiments. The results showed that both PE and an equivalent amount of PU could promote AMPK phosphorylation by decreasing the ATP/ADP ratio in a time-dependent manner, suggesting that PU is the major active component in PE. As a result, ACC was also phosphorylated by PE and PU treatment, indicating the activation of the AMPK pathway. Interestingly, PU’s effects were not universal as this activation was observed only in cardiomyocytes. However, the reason why PU had no effect on neonatal fibroblasts requires further investigation.

AMPK activation works to maintain cellular energy stores by enhancing ATP production, especially through mitochondrial biogenesis, which is required to normalize hyperglycemia-induced ROS production^42^ and prevent HFD-induced lipid accumulation in adipose tissue^43^. By activating the AMPK pathway, PU efficiently promoted mitochondrial biogenesis in cardiomyocytes. In heart tissue, PE supplement showed a preventive effect on the HFD-induced decrease in mitochondria as evidenced by the decreased expression of PGC1α and the complex I, II, III, and V subunits. In addition to mitochondrial biogenesis, mitochondrial network dynamics are also critical to cellular function but have not been thoroughly studied. In the current study, the mitochondrial fusion-related proteins Mfn1 and OPA1 were significantly decreased in the HFD group, suggesting decreased mitochondrial fusion potential, which has been reported to cause endoplasmic reticulum (ER) stress^44^. However, by analyzing ER stress markers, we did not find significant changes (Fig. S4). We speculated that ER stress might arise in a longer HFD feeding condition. On the other hand, mitochondrial dynamics has been indicated closely related to the intrinsic apoptosis pathway^45^, and inhibiting mitochondrial fusion promotes apoptosis^46^. Silencing of either Mfn1 or Mfn2 results in mitochondrial fragmentation and an increase in sensitivity to apoptotic stimuli. In the HFD group, we observed an increase in the levels of pro-apoptotic Bcl-2 members (Bak and Bax) and anti-apoptotic Bcl-2 proteins (Bcl-2 and Bcl-X_L_), as well as cleaved PARP, suggesting excessive activation of the apoptosis (Fig. S5). Moreover, our results showed that cardiac UCP2 and UCP3 expression was upregulated by the HFD, which might lead to the depletion of cellular ATP reserves^47^. PE treatment significantly normalized UCP2 and UCP3 expression and increased ATP content, thereby enhancing the mitochondrial oxidative capacity. Additionally, PE supplementation successfully normalized mitochondrial metabolism and prevented the initiation of apoptosis.

The physiological levels of ROS are important to maintain normal cellular functions, but an overload of ROS could exceed the capacity of the antioxidant system and induce oxidative stress. Recent studies have revealed that Nrf2-mediated phase II enzymes are regulated through the AMPK pathway^16^,^17^,^18^. Our previous study also showed a functional association between AMPK and oxidative stress through the modulation of mitochondrial content and the Nrf2 pathway, as observed by a neuroprotective effect^19^. We measured NQO-1 as an indicator of phase II enzymes and showed that PU could sufficiently increase NQO-1 expression through AMPK in cardiomyocytes *in vitro*. As expect, PE efficiently upregulated the GSH/GSSG ratio, GST and total SOD activities, as well as the expression of NQO-1, HO-1 and MnSOD in the rat heart, thus ameliorating HFD-induced oxidative stress.

In conclusion, our study demonstrated that as the major active component of pomegranate extract, PU is a promising compound for preventing HFD-induced cardiac metabolic disorders and impairment via activation of AMPK and its related signaling pathways. Further experimental and clinical investigations are required to explore the additional mechanisms involved and establish the potential clinical applications.

## Methods

The experiments were approved by School of Life Science and Technology, Xi’an Jiaotong University, and performed in accordance with approved guidelines.

### Animals

Sprague-Dawley (SD) male rats were purchased from the SLAC Laboratory Animal Co. Ltd. (Shanghai, China). All animals were housed in a temperature- (22–28 °C) and humidity- (60%) controlled animal room and maintained under a 12-h light/dark cycle with free access to food and water throughout the experiments. After 1 week of acclimatization, the rats were randomly divided into the following three groups (n = 10 for each group): rats fed a normal diet (Control, 12% kcal fat content), rats fed a high-fat diet (HFD, 45% kcal fat content), and rats fed a high-fat diet with 150 mg/kg/day of PE supplementation (HFD + PE150). After 8 weeks of feeding, the rats were fasted overnight and euthanized. All of the animal procedures were approved by Xi’an Jiaotong University Animal Care and Use Committee. And all the methods were carried out in accordance with approved guidelines.

### Chemicals

Antibodies against β-actin were purchased from Sigma (St. Louis, MO, USA). Antibodies against carnitine palmitoyltransferase I (CPT-1M), NQO-1, MnSOD, PGC-1α, Mfn1, Mfn2, UCP2 and UCP3 were purchased from Santa Cruz Biotechnology (Santa Cruz, CA, USA). Antibodies against ACC, p-ACC (Ser 79), AMPK, and p-AMPK (Thr 172) were purchased from Cell Signaling Technology (Danvers, MA, USA). Antibodies against OPA1 and Drp1 were purchased from BD (Franklin Lakes, NJ, USA). Antibodies against complexes I (39 kDa), II (30 kDa), III (51 kDa), IV (40 kDa) and V (55 kDa) were purchased from Invitrogen (Carlsbad, CA, USA). PE containing 40% PU was produced by Tianjin JF-Natural. PU was purchased from Mansite (Chengdu, China).

### Neonatal cardiomyocytes culture

Primary cultures of cardiomyocytes were prepared as previously described^48^. The ventricles of 1-day-old neonatal SD rats were digested with 0.03% trypsin, 0.03% collagenase, and 20 μg/mL of DNase I. The cardiomyocytes were collected based on their differential adhesiveness. The cells were seeded at a density of 5 × 10^4^ in a 6-well plate. The cells were grown in low glucose DMEM containing 10% FBS, penicillin (50 U/mL) and streptomycin (50 μg/mL) at 37 °C in humid air containing 5% CO_2_. Cardiac fibroblasts were also maintained and subcultured in a 6-well plate for the same treatments. The culture medium was changed every other day. Both PE and PU were dissolved in double distilled water at 10 mg/mL and stored at −20 °C. For cellular treatments, both PE and PU were dissolved in DMEM (with 10% fetal bovine serum) to the appropriate final concentration (100 μg/mL and 250 μg/mL for PE, 100 μg/mL for PU).

### Blood sample preparation

After the rats were euthanized, blood samples were obtained by cardiac puncture, and the serum was separated by centrifugation (1, 200 *g*, 10 min). The serum LD levels as well as the lactate dehydrogenase (LDH) and creatine kinase (CK) activities were determined using commercial kits according to the manufacturer’s instructions (Jiancheng, Nanjing, China).

### Biochemical analysis

Small sections of heart tissue were collected and homogenized in ice-cold phosphate buffered saline (PBS). After centrifugation (1,000 g, 10 min), the supernatant was collected for analysis. GSH, GSSG, triglyceride (TG) and total cholesterol (TC) levels were analyzed using commercial clinical diagnosis kits according to the manufacturer’s instructions (Jiancheng, Nanjing, China). ATP content was also detected in fresh heart tissue using an ATP bioluminescent assay kit (Sigma) as previously described^38^. The activities of glutathione S-transferase (GST), glutathione peroxidase (GPX) and total superoxide dismutase (SOD) in heart tissue were assayed using the Jiancheng Biochemical detection kits according to the corresponding kit protocols. For cellular samples, ATP and ADP contents were analyzed using the BioAssay ADP assay kit, and cellular ATP/ADP ratio was also calculated.

### Protein carbonylation assay

Protein carbonyls in soluble proteins were assayed using the Oxyblot protein oxidation detection kit (Cell Biolabs, San Diego, CA, USA). Protein carbonyls were labeled with 2,4-dinitrophenylhydrazine and detected by Western blot. As a loading control, equal amounts of the samples were subjected to 10% SDS-PAGE and stained with Coomassie brilliant blue.

### Western blotting

Tissue and cellular samples were washed with ice-cold phosphate-buffered saline (PBS) and lysed with Western and IP lysis buffer (Beyotime, Jiangsu, China). The lysates were homogenized and centrifuged at 13,000 g for 15 min at 4 °C. Then, the protein concentrations of the collected supernatants were determined using a BCA Protein Assay kit (Pierce, Rockford, IL, USA). The extracted protein samples (15–20 μg per lane) were subjected to 8%, 10% or 12% SDS-polyacrylamide gel electrophoresis (SDS-PAGE), and the separated proteins were transferred to nitrocellulose membranes (PerkinElmer Life Science, Boston, MA, USA) and blocked with 5% nonfat milk/TBST for 1-2 h at room temperature. The membranes were incubated with primary antibodies at 4 °C overnight, after which they were incubated with horseradish peroxidase-conjugated secondary antibodies for 1 h at room temperature. Western blots were developed using an ECL Western blotting detection kit (Pierce, Rockford, IL, USA) and quantified by scanning densitometry.

### Statistical analysis

The data are presented as the means ± S.E.M. Statistical analyses were conducted using one-way ANOVA followed by LSD post hoc analysis. For all analyses, values of p < 0.05 were considered to be statistically significant.

## Additional Information

**How to cite this article**: Cao, K. *et al*. Punicalagin, an active component in pomegranate, ameliorates cardiac mitochondrial impairment in obese rats via AMPK activation. *Sci. Rep*. **5**, 14014; doi: 10.1038/srep14014 (2015).

## Supplementary Material

Supplementary Information
