# Supplementary material for: Punicalagin, an active component in pomegranate, ameliorates cardiac mitochondrial impairment in obese rats via AMPK activation
Source: Sci Rep. 2015 Sep 15;5:14014. doi: 10.1038/srep14014 (PMC4642696; doi:10.1038/srep14014)
Supplement: Supplementary Information [file srep14014-s1.pdf]

Supplementary materials

Punicalagin, an active component in pomegranate, ameliorates cardiac mitochondrial impairment in obese rats via AMPK activation

Ke Cao[1], Jie Xu[2], Wenjun Pu[2], Zhizhong Dong[3], Lei Sun[4], Weijin Zang[4], Feng Gao[5], Yong Zhang[2, 6], Zhihui Feng[1*], Jiankang Liu[2*]

[1]Center for Mitochondrial Biology and Medicine, Frontier Institute of Science and Technology, Xi'an Jiaotong University, Xi'an, 710049, China; [2]The Key Laboratory of Biomedical Information Engineering of Ministry of Education, School of Life Science and Technology, Xi'an Jiaotong University, Xi'an, 710049, China; [3]Nestlé Research Center Beijing, Beijing, 100095, China; [4]Department of Pharmacology, School of Medicine, Xi'an Jiaotong University, Xi'an, 710049, China. [5]Department of Physiology, Fourth Military Medical University, Xi'an, 710032, China; [6]Tianjin Key Laboratory of Exercise Physiology and Sports Medicine, Tianjin University of Sport, Tianjin, 300381, China.

Word count: 4,995; **References:** 51

Greyscale illustrations: 8; **Color illustrations:** 0

Running title: AMPK activation by punicalagin in hearts of obese rats

***Corresponding authors:**

Zhihui Feng, PhD and Jiankang Liu, PhD

Center for Mitochondrial Biology & Medicine

Xi'an Jiaotong University

28 W. Xian-ning Road

Xi'an 710049, China

Tel: 86-29-82664232

E-Mail: zhfeng@mail.xjtu.edu.cn and j.liu@mail.xjtu.edu.cn

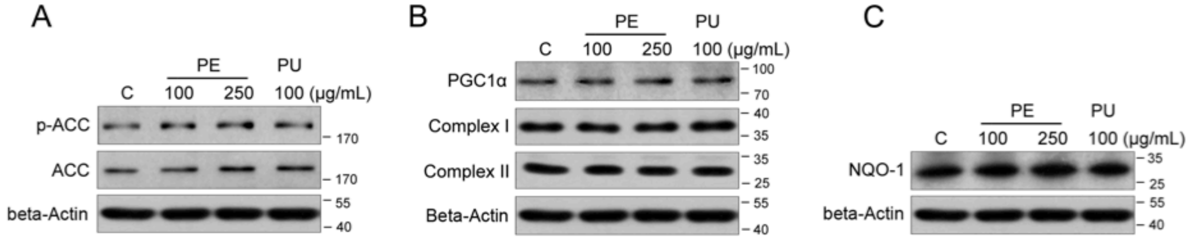

Supplementary Figure 1. Effects of PU and PE on AMPK pathway in neonatal cardiac fibroblasts. (A) Neonatal cardiac fibroblasts were treated with PE at 100, 250 μg/mL and PU at 100 μg/mL for 2 h, p-ACC and ACC was analzyed by western blot. Neonatal cardiac fibroblasts were treated with PE at 100, 250 μg/mL and PU at 100 μg/mL for 24 h, mitochodnrial biogenesis related PGC-1α, complex I, II (B), and phase II enzyme NQO-1 (C) was analzyed by western blot.

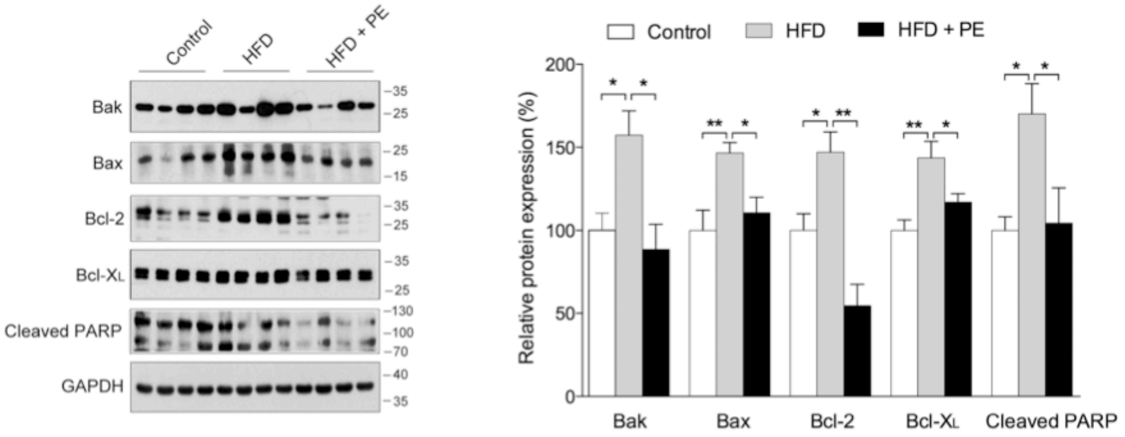

Supplementary Figure 2. PE and HFD on cardiac apoptosis pathway. SD rats in the HFD groups were administered either saline or PE (150 mg/kg/day) for 8 weeks. Protein samples were prepared from heart tissue. Proteins in apoptosis pathway were analyzed by western blot (A: blot image; B: arbitrary unit analysis). The values are presented as the means ± S.E.M. (n ≥8); *p<0.05, **p<0.01.

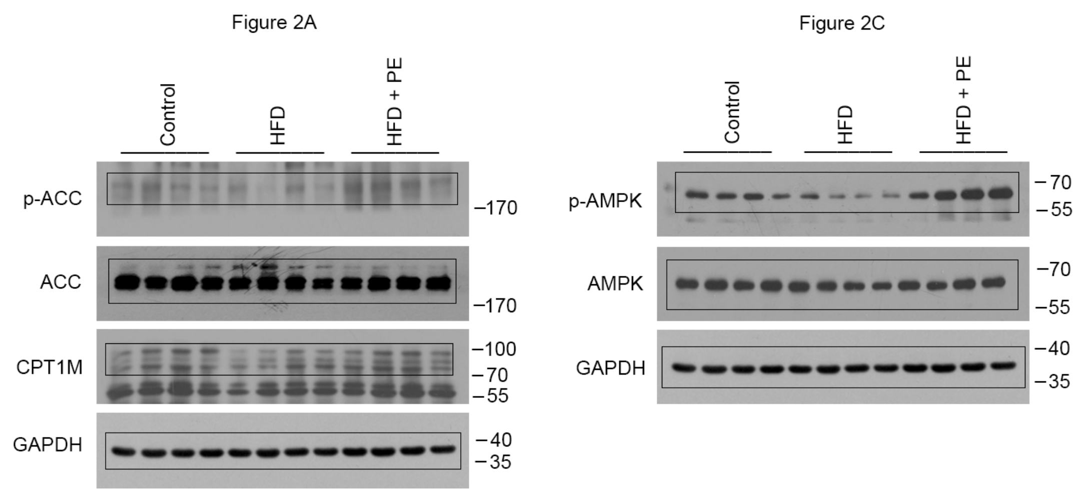

Supplementary Figure 3. Uncropped Western blots to the corresponding figures. Molecular weight makers are indicated in KDa.

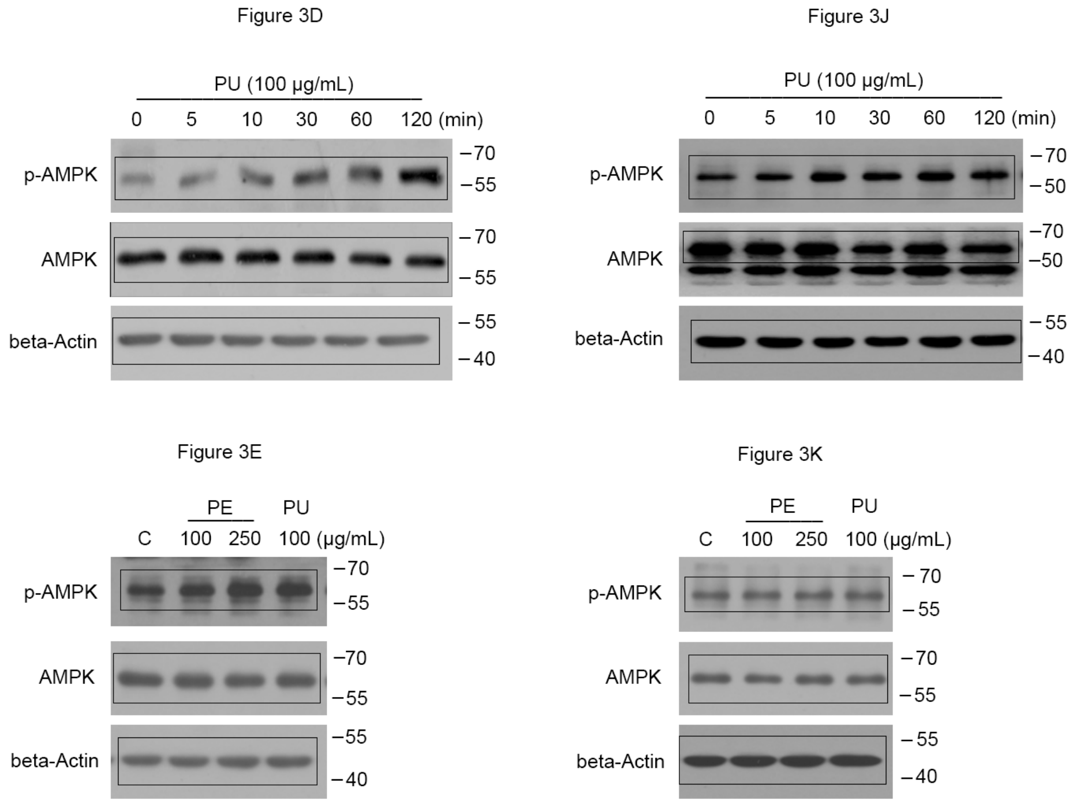

Supplementary Figure 4. Uncropped Western blots to the corresponding figures. Molecular weight makers are indicated in KDa.

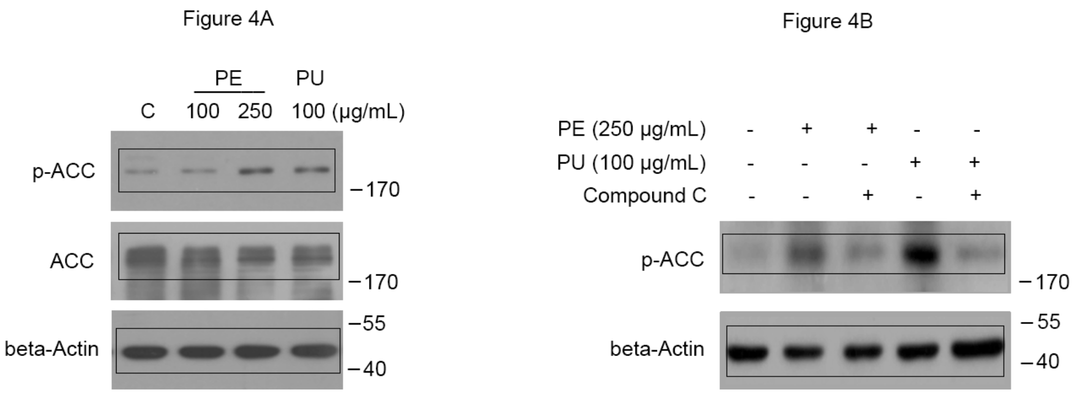

Supplementary Figure 5. Uncropped Western blots to the corresponding figures. Molecular weight makers are indicated in KDa.

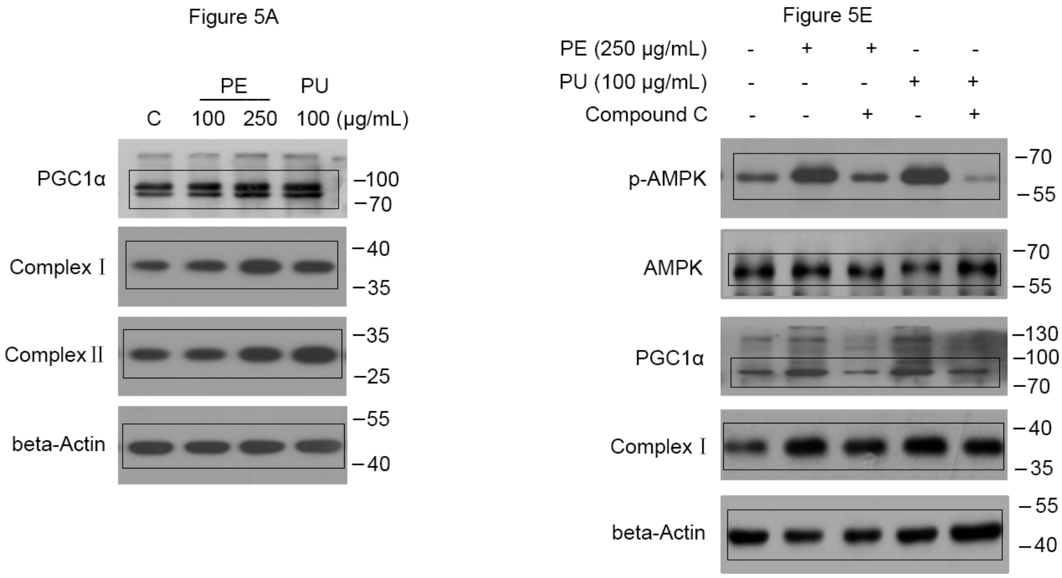

Supplementary Figure 6. Uncropped Western blots to the corresponding figures. Molecular weight makers are indicated in KDa.

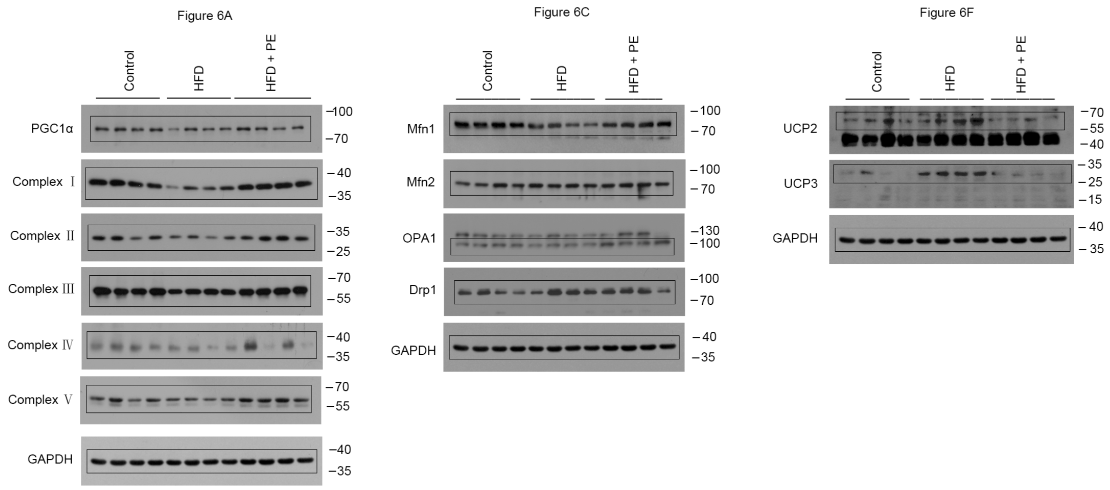

Supplementary Figure 7. Uncropped Western blots to the corresponding figures. Molecular weight makers are indicated in KDa.

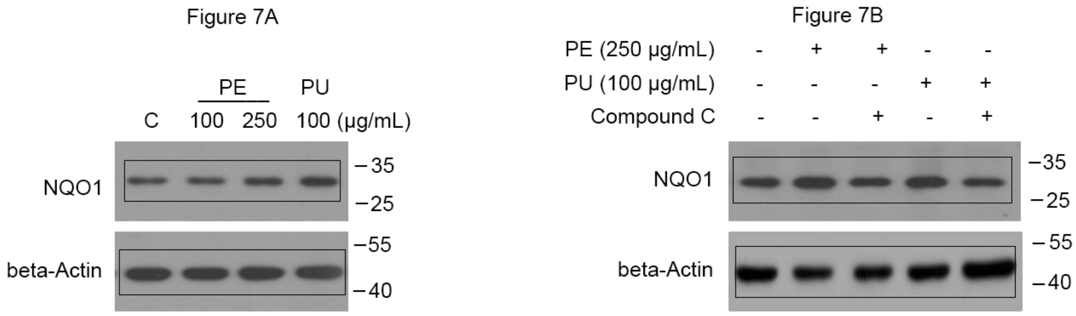

Supplementary Figure 8. Uncropped Western blots to the corresponding figures. Molecular weight makers are indicated in KDa.

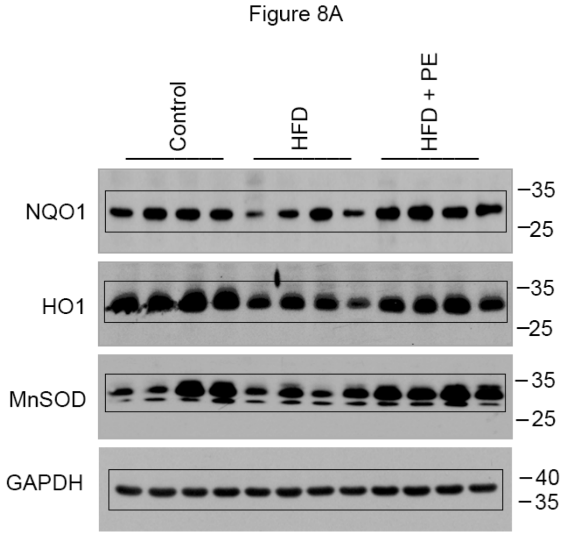

Supplementary Figure 9. Uncropped Western blots to the corresponding figures. Molecular weight makers are indicated in KDa.
